# Approach to loose teeth: An alternative solution

**DOI:** 10.4103/0019-5049.63640

**Published:** 2010

**Authors:** Nishkarsh Gupta, Pradeep Karunagaran, Mridula Pawar

**Affiliations:** Department of Anaesthesiology and Intensive Care, P.G.I.M.E.R, Dr. Ram Manohar Lohia Hospital, New Delhi, India

Sir,

A loose tooth or teeth always pose a problem for the anaesthesiologist during laryngoscopy and endotracheal intubation. This problem is aggravated if the loose tooth happens to be one of the upper incisors, especially in anticipated difficult intubation. We face this problem more frequently because of poor oro-dental hygiene in the patients.

Preoperative extraction is one possibility during planned surgery. A few other measures have been tried to avoid trauma to the loose teeth and to prevent their accidental dislodgement into the airway. Singhal[1] *et al*. have tried passing a silk thread across the loose tooth through the interdental cleft and tying the knot at the base of the tooth.

In case of the loose teeth being the upper incisors, we felt that a molar approach to intubation would avoid contact with the loose teeth. The left molar approach to intubation with optimal external laryngeal manipulation (OELM) has been found to improve the laryngeal view in patients with difficult laryngoscopy and has been found to offer a better laryngoscopic view than a right molar approach.[2]

We have tried the left molar approach to intubation using a Macintosh 3 blade in 10 patients with loose upper incisors. The procedure was explained to all the patients and written informed consent was taken from them. We were able to successfully intubate all the patients in the first attempt with the use of stylet along with OELM. With this approach, the possibility of trauma to the loose teeth and their dislodgement is eliminated. We therefore feel that the left molar approach to intubation is a safe and effective method of securing the airway in patients with loose upper incisors.
